# Genetic Aspects of Inflammation and Immune Response in Stroke

**DOI:** 10.3390/ijms21197409

**Published:** 2020-10-08

**Authors:** Dejan Nikolic, Milena Jankovic, Bojana Petrovic, Ivana Novakovic

**Affiliations:** 1 Faculty of Medicine, University of Belgrade, 11000 Belgrade, Serbia; ivana.novakovic@med.bg.ac.rs; 2 Physical Medicine and Rehabilitation Department, University Children’s Hospital, 11000 Belgrade, Serbia; 3 Neurology Clinic, Clinical Center of Serbia, 11000 Belgrade, Serbia; milena.jankovic.82@gmail.com; 4 Clinic for Gynecology and Obstetrics, Clinical Center of Serbia, 11000 Belgrade, Serbia; bojana.petrovic1977@gmail.com

**Keywords:** genetics, inflammation, stroke, treatment, recovery

## Abstract

Genetic determinants play important role in the complex processes of inflammation and immune response in stroke and could be studied in different ways. Inflammation and immunomodulation are associated with repair processes in ischemic stroke, and together with the concept of preconditioning are promising modes of stroke treatment. One of the important aspects to be considered in the recovery of patients after the stroke is a genetic predisposition, which has been studied extensively. Polymorphisms in a number of *candidate genes*, such as *IL-6*, *BDNF*, *COX2*, *CYPC19*, and *GPIIIa* could be associated with stroke outcome and recovery. Recent GWAS studies pointed to the variant in *genes* *PATJ* and *LOC* as new genetic markers of long term outcome. Epigenetic regulation of immune response in stroke is also important, with mechanisms of histone modifications, DNA methylation, and activity of non-coding RNAs. These complex processes are changing from acute phase over the repair to establishing homeostasis or to provoke exaggerated reaction and death. Pharmacogenetics and pharmacogenomics of stroke cures might also be evaluated in the context of immuno-inflammation and brain plasticity. Potential novel genetic treatment modalities are challenged but still in the early phase of the investigation.

## 1. Introduction

Stroke is a complex disease with a substantial genetic component, the heritability of which ranges from 16% to 40% [1]. Around 85–90% of all stroke cases account for ischemic stroke (IS) due to an embolus or thrombosis causing vascular occlusion in situ in certain brain parts; in the rest of cases, hemorrhagic stroke (HS) occurs [2].

Cerebrovascular insult initiates a complex cascade of events at genomic, molecular, and cellular levels, and inflammation is important in this cascade, both in the Central Nervous System (CNS) and in the periphery [3]. Genetic aspects of inflammation and immune response in stroke cover several fields that will be discussed in this paper.

## 2. Inflammation and Immunomodulation as Treatment Modes in Stroke

Aside from beneficial effects of inflammation in certain conditions, when its actions are prolonged they can lead to less favorable outcomes. Systemic inflammation as well as inflammation in the brain is associated with neuronal loss in stroke patients [4]. However, besides the negative effects, inflammation might be associated with repair processes in ischemic stroke [5]. One of the important factors for both of these effects is time. Jian et al., stated that in the early stage after the stroke, microglia are recruited, while peripheral immune cells appear within one day (neutrophils even within the first hour) and lasting until seven days after the stroke event [6]. The CD8^+^ T cells appear 3 h after the stroke at earliest, while CD4^+^ T cells within 24 h after the stroke. The dynamics of immune cells appearance is important, having in mind especially their potential role in stroke, where neutrophils produce inflammatory factors and by the mechanisms of phagocytosis might promote tissue healing, while CD8^+^ T cells can be associated with the neuronal death and CD4^+^ T cells might play the role in tissue repair [6]. In the mice model, depletion of cytotoxic T lymphocytes had favorable effects on infarct volumes and behavioral deficits [7]. Moreover, the subpopulation of T cells or regulatory T cells (Treg) are endogenous modulators with potential neuroprotective effects, where the anti-inflammatory cytokine *IL-10* plays important role in their function by augmenting Treg’s or their downstream signaling pathways [6,8]. They appear a few days after the stroke and persisting more than 30 days [9]. The specific phenotypes of microglia or macrophages are also important when assessing post stroke tissue damage, where the M1 population is considered to be predominantly destructive, while the M2 population neuroprotective [10]. Schuhmann et al., in their study stressed that the B cells in the acute phase of the induced stroke in animal models do not influence on lesion volume and functional outcome [11].

During the acute phase that can last from minutes to hours [12], damaged brain cells can stimulate systemic immunity by releasing the specific signals that could lead to the immunodepression, thus increasing the risk of potential infections [13]. Additionally in this stage, there is the process of the promotion of adherence and transendothelial transfer of leukocytes [12]. Further, in the subacute phase the production of matrix metalloproteinases (MMP) is noticed, leading to the numerous unfavorable effects, including disruption of the blood-brain barrier, brain edema, and neuronal death [12]. In the chronic phase, adaptive immune response affecting the brain might increase the possibility of poststroke morbidity [13]. It was shown that in mice with induced cerebral ischemia there is an increased risk of infections, particularly pneumonia [9].

Additionally, patients with stroke also have a certain degree of immunosuppression that could have neuroprotective effects via cytokines and growth factors [5]. In the study of Li et al., it was noticed that for patients with acute ischemic stroke, serum cytokines are associated with stroke severity and cerebral infarct volume, particularly with *IL-5* as an independent protective factor for prognosis [14]. Protective effects of *IL-4* on animal models probably by reduced inflammation were addressed in the study of Xiong et al. [15]. Furthermore, promising results were noticed with antagonist *IL-1ra* in the treatment of acute ischemic stroke, where patients had a greater reduction in NIH stroke scale scores versus those who were administered a placebo for at least 3 months after the treatment [16].

In summary, it can be postulated that by controlling the inflammation and immune responses, the brain tissue damage might be controlled to a certain degree as well, along with potential improvements in stroke outcome. Therefore, large and well-designed clinical trials with standardized methods are needed in future research.

## 3. Preconditioning in Stroke

The phenomenon of preconditioning or ischemic tolerance was described in several previous studies [17,18,19]. In the study of Anrather et al., authors pointed that this phenomenon might alter the tolerance of the entire organism to a more lethal stimulus by previously applying stressful but sublethal stimulus probably by a cascade of molecular and biochemical events [19]. Two types of tolerances were proposed and described: the rapid tolerance lasting for a few hours and delayed tolerance probably associated with the new gene expression along with de novo protein synthesis [20]. The neuroprotective effects of preconditioning might be explained by a complex cascade of signaling events that are leading to new protein synthesis, a process proposed as a genomic reprogramming model [20]. Additionally, the increase of intragenic methylation is described in a model of preconditioning ischemia [21]. Moreover, mitochondrial roles in preconditioning were studied as well. Particularly, protective effects of the integrity of mitochondrial oxidative phosphorylation, and preserving mitochondrial function in tested subjects with cerebral ischemia, were seen for delayed preconditioning [22]. Therefore, the preconditioning as a phenomenon with protective effects in stroke sufferers might be considered in the future as one of the promising potential treatment modes.

From an evolutionary point of view, stroke is affecting the two most important systems for survival (nervous and immune), it’s usually happening after the reproduction phase and it’s not compatible with life in the wilderness [23]. Having that in mind, it is possible that the immune system is overreacting and not trying to establish homeostasis. As previously mentioned, the preconditioning immune system could “learn” that the living after a stroke is possible and to react in more helpful manner [20]. Another approach is the development of therapies for balancing immune reaction, more precisely, shifting microglial activity from pro-inflammatory and neurotoxic to anti-inflammatory and neuroprotective.

## 4. Genetics and Inflammation in Post Stroke Recovery

The importance of recovery in the post-stroke period increased in recent years especially with the advancement of medicine, science, and technology. The major burden in stroke survivors is a disability that affects various dimensions of daily living and quality of life. This disability is presented with wide variability among individuals in post-stroke period. Early diagnostics with adequate triage and implementation of innovative treatment protocols are considered to be very important in early recognition and controlling the progress and dynamics of tissue damage in affected areas of the brain [24,25,26,27]. Once the disability is present, post-stroke survivors are included in rehabilitation treatment for optimal functional improvements and prevention of further functional decline [28,29]. Nowadays, special consideration is given to promising interventions including virtual reality in the rehabilitation and telerehabilitation of stroke affected patients [30,31,32]. Furthermore, patient-tailored treatment along with personalized management in the post-stroke period will hopefully bring to better health care and quality of life for these patients.

One of the important aspects to be considered in the recovery of patients after the stroke is a genetic predisposition as well as potential novel genetic treatment modalities. Genetics of Ischaemic Stroke Functional Outcome Network (GISCOME) was established for identification of potential genetic loci that might influence the functional outcomes in stroke survivors [33]. So far it was pointed that genetic factors could be associated with long-term stroke outcomes, thus further genetic analyses might bring new insights for better understanding of molecular mechanisms regarding the stroke outcome [1]. Considering the acute and subacute stroke outcomes, the early neurological deterioration is found potentially to be multifactorial, with different single nucleotide polymorphisms (SNPs) that are independently associated with such condition [1,34,35]. 

In the meta-analysis of Math et al., it was noticed that variants of *BDNF* and *CYP2C19* genes are negatively associated with the recovery of patients after ischemic stroke, while the *APOE4* gene was shown to be negatively associated with recovery of intracerebral hemorrhage [36]. Furthermore, these authors pointed that some genes involved with the drug metabolism including *COX2*, *MRD1* as well as *CYP2C19*, possibly by inflammatory cascade, might have certain roles in the recovery after acute ischemic stroke [36]. The study of Maguire et al., demonstrated as well that 2 *COX2* variants (rs20417 and rs5275), as well as *GPIIIa* variant (rs5918), are associated with functional outcomes in stroke survivors [37].

Previous reports addressed that immune response and inflammation are important factors that are associated with stroke pathogenesis and its outcome [19,38]. Even though inflammation starts locally, its mediators are disseminated inducing systemic inflammatory response [19]. The released proinflammatory mediators by vascular endothelium and brain parenchyma might locally increase the injury of the affected tissue in a stroke event. Chakraborty et al., conducted the study on genetic analyses of *IL-6* gene promoter polymorphism and stated that a GC genotype in the study group who suffered a stroke had significantly higher levels of *IL-6* versus those of CC and GG genotypes. Furthermore, the group with GC genotype was shown to have poorer short-term as well as a long-term outcome [39]. Increased levels of *IL-6* were previously described in patients with acute ischemic stroke. This pro-inflammatory cytokine could induce excessive inflammatory response affecting the injury pathogenesis in stroke patients leading to a less favorable outcome [38]. The correlation of *IL-6* and stroke outcome was shown in the study of Aref et al., where it was stated that the worse outcome 3 months post-stroke event was in subjects with higher levels of *IL-6* [40]. Additionally, the recurrence of stroke was also associated with higher levels of *IL-6* [40].

Several genome-wide association studies (GWAS) have found genes associated with stroke risk and results have been confirmed in independent studies [41,42]. However, GWAS studies performed to find genetic variables associated with stroke outcomes are not so frequent. Recently, about long-term stroke outcome, two GWAS studies have been published with remarkable results. The GODS (Genetic contribution to functional Outcome and Disability after Stroke) study detected that PATJ (Pals1-associated tight junction) low-frequency variants were associated with worse IS functional outcome [43]. In the GISCOME study, with more than 6000 participants, it was showen that intronic variant rs1842681 in LOC105372028 had a significant association with functional outcomes 60–90 days after stroke [44]. It is important to emphasize that the *LOC* gene is involved in the expression of protein phosphatase 1, which is implicated in brain plasticity. Several other variants, some within or near genes that have been linked to outcomes in animal models of stroke, also had a suggestive association with patient outcomes, but under the range of significance [44]. These studies are beginning to clarify the influence of genetics on patient recovery, which can help us to understand all the mechanisms involved.

## 5. Epigenetic Regulation of Immune Response in Stroke

Completely simplified, we can consider stroke as an acute and devastating vascular insult, causing oxygen-glucose deprivation (OGD) and leaving high-energy-demanding brain cells in metabolic stress. Such dramatic consequences require: (1) prompt response from local immune cells, (2) information spreading to other parts of the brain and the whole organism and (3) reactions in order to recover and, if possible, to re-establish homeostasis. The fastest way to change the modality of cell metabolism and signaling is trough epigenetic modifications on transcriptional and post-the transcriptional levels. Epigenetic traits are defined as heritable changes in gene expression emerging from interactions between the environment and the genome, although without alterations in the DNA sequence [45]. The most prominent epigenetic mechanisms that have been addressed in human and animal studies of immune response and stroke are histone modifications (acetylation, methylation, etc.), DNA methylation and non-coding RNAs gene expression regulation.

### 5.1. Histone Modifications

Post-translational, reversible, and extremely sensitive modifications of histones, are affecting DNA wrapping around histones in nucleosome formation and chromatin packing and organization [46]. Histone acetylation/deacetylation is regulated by two groups of enzymes (histone acetyltransferases (HATs) and histone deacetylases (HDACs)) and generally increases or inhibits transcriptional activation, respectively [47]. It has been widely reported that stroke is provoking histone deacetylation and that HDAC inhibitors are usefully reducing the process. Those inhibitors also suppressed the expression of proinflammatory proteins and connected drugs administration with changes in microglia in animal models of stroke [48]. Valproic acid is reducing inflammation, disruption of the blood-brain barrier (BBB), and improving outcome trough HDAC inhibition in rat models of hemorrhage or ischemic stroke [49,50,51]. Recent studies demonstrated that inhibition of HDAC with sodium butyrate is upregulating expression of anti-inflammatory mediators *IL-10* and *STAT3*, and suppressing the expression of pro-inflammatory mediators, *TNF-α* and *NOS2*, *IL-1* and *IL-18* [52,53]. Additionally these results suggested the biphasic effect of sodium butyrate, initially suppressing inflammation by reduction of BBB permeability, and later, promoting recovery by elevation of *IGF-1* expression in peripheral tissues [52]. Methylation is another form of histone modification, catalyzed by histone methyltransferases (HMTs) [54]. Its role is complex and may differentially affect gene expression, depending on temporal and spatial changes on the tissue level. Studies of animal and in vitro models of stroke demonstrated that histone methylation is affecting stroke severity during aging [55] and neuronal resistance in a model of hypoxic metabolic stress [56]. Besides that, studies implicated the connection of histone methylation with proinflammatory cytokines [57].

### 5.2. DNA Methylation

DNA methylation is defined as the attachment of methyl (CH3) group to a 5′ position of the cytosine ring. The formation of 5-methylcytosine (5-mC) does not affect all cytosine residues in the DNA, rather only palindromic sites, named CpG islands. Those sites are usually located near or in promoter regions allowing the family of enzymes, DNA methyltransferases (DNMTs), to regulate gene function trough methylation process [58]. DNA methylation is preventing transcription factors binding, and, in most cases, decreasing transcription, but depending on tissue microenvironment and position in the genome, it can also promote gene expression [59]. Beside the methylation of promoter regions, alteration in gene body methylation is recognized as regulatory mechanism of gene expression and recent study confirmed that DNA methylation of exons is involved in alternative splicing [60]. Recent evidence also shed light on interaction between allele-specific DNA methylation (ASM), allele-specific binding of transcription factors (ABTF), genetic variants and environmental factors, and their role in disease risk [61]. This is extremely important for better understanding pathogenic mechanisms in multifactor diseases such as stroke.

As one of the most intensively studied epigenetic mechanisms, through a combination of genome wide association studies and a single-cell approach, DNA methylation is established as stable, but dynamic and reversible [59] and in the context of the immune system a very useful mechanism [62]. Early studies on DNMT-deficient mice models have shown an association between general suppression of DNA methylation and resistance to stroke, or covertly that DNA methylation contributes to large tissue damage even after mild ischemic brain injury [63]. Recent studies demonstrated alterations in methylation patterns trough different stages of immune reaction cascade during and after stroke. Gallego-Fabrega et al. analyzed almost 500000 DNA methylation sites in patients treated with antiplatelet drugs (aspirin or clopidogrel). They have revealed the involvement of hypomethylation of 2 “inflammation control” genes: *protein phosphatase 1A (PPM1A)* and *TNF receptor-associated factor 3 (TRAF3)* with increased stroke recurrence [64,65]. In later phases of the immune response, hypermethylated promoter of *thrombospondin-1* (*THBS1*), a gene associated with angiogenesis and neuroprotection, decreases stroke recovery [66,67].

### 5.3. Non-Coding RNAs

Non-coding RNAs are a wide group of regulatory RNAs that are not translated into proteins. Most extensively studied are microRNAs (miRs) and long non-coding RNAs (LncRNAs). MiRs are evolutionarily conserved oligonucleotides with less than 200 nucleotides in length (usually about 20) which are complementary to 3′-UTR of different messenger RNAs (mRNAs). Pairing miR with targeted mRNA is inhibiting translation and leading to mRNA degradation [68]. During the past decade, the role of miRs has been widely addressed in the regulation of stroke consequences, particularly in activation of microglia and the affection of numerous pro- and anti-inflammatory factors following stroke [69]. MiRs are recognized as potential novel therapeutic targets in stroke, but their expression is highly tissue or disease state-dependent. LncRNAs are consisted of more than 200 nucleotides, although, less abundant compared to miRs they show more microenvironment-dependent function [70] and have regulatory roles in a variety of metabolic processes in the nucleus and cytoplasm [71]. In recent years, the function of different lncRNAs in expression regulation after stroke is studied more extensively, especially in inflammation [72,73,74]. Interestingly, lncRNA may regulate the expression of genes coding for miRs. The negative correlation of nuclear enriched abundant transcript 1 (*NEAT1*) lncRNA has been shown in stroke prognosis and recovery with miR-124 and miR-125a, which are identified as inflammatory miRs [75].

**Figure 1:**
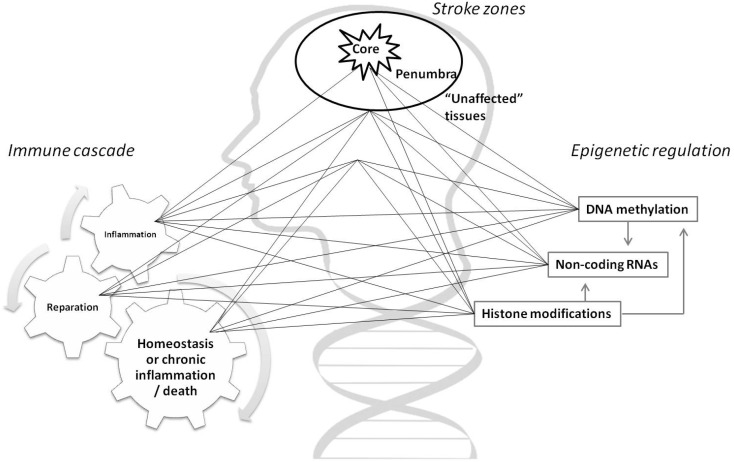
Spatio-temporal pattern of epigenetic regulation in immune response in stroke. The scheme is highlighting multidimensional relations of the immune system and epigenetic mechanisms in different stroke zones over time. Stroke is unequally affecting brain tissues, with irreversible neuronal damage in stroke core and metabolic changes with the possibility counteract tissue injury in the penumbra area. Additionally, stroke is provoking immune response leading to inflammation and starting an immune cascade with consequences not only in the brain but also in the whole organism. Alteration of immune function is conducted through complex epigenetic regulation which is sensitive to temporal changes in the tissue microenvironment.

It would be important to mention that complex epigenetic regulation of immune response in stroke is changing from the acute phase (inflammation), over repair phase to establishing homeostasis, or to provoke exaggerated reaction and death [76]. Contribution to complexity gives spatial distribution of different processes in stroke core, penumbra or unaffected tissues with numerous, overlapping regulatory layers (Figure 1). An increasing number of studies will contribute to more detailed maps of the epigenomic landscape, but its dynamic signature is complicating attempts to define useful biomarkers and predict the outcome of prevention measures and potential therapies.

## 6. Pharmacogenetic Markers in Stroke 

The ability of a brain to repair after a stroke depends on certain factors. Therapeutic options in stroke are turned towards the regeneration of a brain cell network taking into account neuroplasticity. Despite pharmaceutical efforts to help this process, pharmacogenomics gave evidence about resistance to stroke therapy due to individual variability in response to the drug.

In IS, nerve cells in the central zone of ischemia cannot be saved. However, in the surrounding penumbra neurons are functionally disturbed, but structurally intact, with the possibility for the recovery of their function. Without timely recanalization, an irreversible structural and functional damage in the zone of penumbra will occur with a significant effect on neurological damage and disability level [77]. The therapy of choice in the acute phase of IS is an intravenous administration of recombined tissue plasminogen activator (rt-PA). However, this therapy can cause potentially serious side effects, mostly hemorrhagic complications and the administration criteria are strictly defined having in mind inter-patient variability [78]. Pharmacogenetic investigations in this field are at the beginning, with available results from only several studies. The strong candidate gene in such studies is *PAI-1* (plasminogen activator inhibitor 1). The product of the *PAI-1* gene binds to t-PA, forming an inactive complex and acting as an inhibitor of endogenous fibrinolytic activity [79]. According to the literature data, only a few studies considered the association between *PAI-1 4G/5G* gene polymorphism and rtPA efficacy, with conflicting results. Fernandez-Cadenas revealed that the 4G/4G genotype may be responsible for the poorer recovery of patients with IS treated with rtPA [80]. A recent study by Dusanovic Pjevic et al. could not confirm the impact of 4G/5G polymorphism on patient’s recovery evaluated by the modified Rankin scale (mRS), nor its influence on the rate of hemorrhagic transformation (HT) [81]. Another candidate gene is *ACE* (*angiotensin-converting enzyme*) because *ACE*, beside the vasoconstrictive effects, has a role in fibrinolysis suppression. However, the importance of *ACE I/D* gene polymorphism in pharmacogenetics of rt-PA and stroke outcome is still controversial [81].

Antiplatelet drugs are widely used in stroke therapy and prevention so their pharmacogenetics and pharmacogenomics are extensively analyzed. Studies revealed single nucleotide polymorphisms (SNPs) of cytochrome P450 (CYP) enzymes and their interactions affect biotransformation of antiplatelet drug Clopidogrel, inducing resistance to therapy in acute ischemic stroke [82,83]. Genetic variants and specific gene-gene interactions among cyclooxygenase-2 (*COX-2*), platelet membrane receptor P2Y1 and glycoprotein *GPIIIa* are associated with aspirin resistance. Interestingly enough, the same gene variants could be associated with early neurological deterioration, as it is described above [32]. Oral anticoagulant therapy also showed clear pharmacogenetic markers responsible for interindividual dosage variability and linked to the risk of bleeding as an adverse effect. Studies confirmed that *CYP2C9* and *VKORC1* (vitamin K epoxide reductase complex, subunit 1) gene variants constitute strong risk factors of warfarin-related intracerebral hemorrhage. In addition, there are data about the role of both ε2 and ε4 variants in the *APOE* gene as risk alleles. [84,85,86]. 

The novel epigenetic and sequencing studies should be designed to examine the pharmacogenetics of the drug resistance in stroke, as the essential part of personalized medicine.

## 7. Gene Therapy for Stroke

One of the main scientific efforts in the field of stroke gene therapy is to genetically reprogram cells in order to reduce the inflammatory response and to initiate regeneration of damaged tissue during stroke recovery. A new gene therapy turns glial cells into neurons, repairing the damage that results from stroke and significantly improving motor function in rodents.

Zeng et al. showed that the tripartite motif containing 9 (TRIM9), a brain-specific ubiquitin ligase, is a potent inhibitor of a *nuclear factor kappa* (NF-κB) signaling pathway in cell culture in vitro (upon cytokine stimulation). In murine stroke models, systemic administration of a recombinant adeno-associated virus that drove brain-wide TRIM9 expression effectively resolved neuroinflammation and alleviated neuronal death (especially in aged mice). Their findings suggest that TRIM9 is essential for resolving NF-kB-dependent neuroinflammation to promote recovery and repair after brain injury and that manipulating TRIM9 expression may represent an attractive immunomodulatory therapeutic target [87].

G-CSF (granulocyte-colony stimulating factor) is an endogenous ligand in the CNS that displays several important functions including anti-apoptotic activity, immunomodulatory action, stimulates neurogenesis, and angiogenic capabilities. *G-CSF* treatment exerts neuroprotective effects on damaged neurons by decreasing pro-apoptotic proteins and increasing of antiapoptotic proteins, both reducing acute neuronal degeneration and adding to long-term plasticity after cerebral ischemia [88,89]. Balseanu et al., demonstrated in a stroke rat model that daily intravenous injection of *G-CSF* led to robust and consistent improvement of neurological functions, which in a combination with a single intravenous administration of mesenchymal stromal cells (MSC) increased the neurogenesis in the subventricular zone and improved microvessel density in the formerly infarct core and perilesional area of treated aged rats, but had no beneficial effect on the infarct volume or mortality [90]. The same group revealed that the combination of *G-CSF* with bone marrow-derived mononuclear cells (BM MNC) in aged rats led to the no advantage over *G-CSF* treatment alone, suggesting that different outcomes might be achieved depending on the type of cells used [91]. Ren et al. confirmed the neuroprotective effect of *G-CSF* gene therapy in rodents and suggested a translational possibility of this research strategy in humans by exprimation of the *G-CSF* gene into an adenovirus that is safe and known to infect brain cells efficiently [92].

Chen et al. identified leucine zipper-bearing kinase *LZK* as a critical cell-intrinsic regulator of astrocyte reactivity, and consequently post-injury recovery and repair of the mammalian CNS. Using genetic loss-of-function and gain-of-function strategies in vivo, they showed that the conserved *LZK* promotes astrocyte reactivity and glial scar formation after CNS injury. Induced *LZK* gene deletion in astrocytes of adult mice reduced astrogliosis and impaired glial scar formation, resulting in increased lesion size after spinal cord injury. Conversely, *LZK* overexpression in astrocytes enhanced astrogliosis and reduced lesion size. Remarkably, in the absence of injury, *LZK* overexpression alone induced widespread astrogliosis in the CNS and upregulated astrogliosis activators *pSTAT3* and *SOX9*. These results enable broad translational implications for neural repair [93].

Results obtained by Sokolov et al. imply that intrathecal injection of genetically engineered umbilical cord blood mononuclear cells (UCB-MC) over-expressing therapeutic molecules vascular endothelial growth factor (VEGF), glial cell line-derived neurotrophic factor (GDNF), neural cell adhesion molecule (NCAM) might represent a novel avenue for future research into treating stroke. Remodeling of the brain cortex in the stroke area was confirmed by the reduction of infarct volume and attenuated neural cell death, depletion of astrocytes and microglial cells, and an increase in the number of oligodendroglial cells and synaptic proteins expression [94].

NeuroD1 (neurogenic differentiation 1) is a member of the NeuroD family of basic helix-loop-helix transcription factors. Chen et al. were reported regeneration of one third of the total lost neurons after ischemic injury and simultaneously protection of another one third of injured neurons, leading to a significant neuronal recovery, using NeuroD1 adeno-associated virus (AAV)-based gene therapy. They demonstrate that in vivo astrocyte-to-neuron conversion mediated through such gene therapy can efficiently regenerate a large number of functional neurons in an ischemic injury model and achieve the functional rescue of both motor and cognitive deficits in rodent animals [95].

Although the effectiveness of gene therapy for stroke is shown only in ischemic stroke animal models, results are highly encouraging and could make a real difference in the future.

## 8. Aging and Inflammation in Stroke

The impact of aging on immune response in stroke patients is of great importance, since majority of them belong to the group of people above 65 years [96,97]. Previously it was stated that the aging, as a non-modifiable risk factor, have a negative impact on the immune response [96]. The studies on animal models revealed that aged mice have reduced infarct volumes but worse functional outcomes. One of possible mechanisms that might explain to the certain degree increased mortality in aged animals after the stroke is a profound splenic contraction affecting peripheral immunosuppression [97]. Additionally, ageing is associated with the decreased brain plasticity, where in aged rats there is a delay of plasticity-associated proteins expression in affected region of the stroke [98]. Popa-Wagner et al., stated that aged animal models have rapid formation of glial scars after the stroke. Such phenomenon might be explained by the premature cellular proliferation, with potential role of nestin positive cells that arise from the capillary wall [99]. Furthermore, it was suggested that nestin, which is neuroepithelial marker, could facilitate cellular structural remodeling after the stroke [100]. Moreover, increased low-grade chronic inflammation is associated with an aging [101]. Further, it can be postulated that in aged individuals presence of chronic systemic inflammation prior to the stroke event could create a “primed” inflammatory environment that might lead to the exacerbation of the post-stroke inflammation response [102]. Therefore, reduced functional recovery potential in aged animal models after the stroke could be associated by the dysregulation in timing and intensity of cellular and genetic responses to the injured tissue [99,103]. 

Aged individuals activate most growth-promoting genes at later time-points following stroke than young adults [103]. In experimental models, inflammatory response of aging brain to ischemia–reperfusion injury is characterized by increased chemokine expression, cytokine expression including *TNF-α*, *IL-1*, *IL-6* and increased cell death. At transcriptional level, five inflammation-related genes in the penumbra region (Protein tyrosine phosphatase receptor type C-Ptprc, Prostaglandin E synthase 3-Ptges3, transforming growth factor, beta receptor I-Tgfbr1, *IL-6*, ribosomal protein *S2-Rps2*) were found up-regulated in aged rats but not in young animals [102]. Regarding testing of new therapeutic options, Popa-Wagner’s group showed the specific response of a aged animals with stroke, as it is previously discussed [90,91]. 

## 9. Conclusions

In the conclusion, studying of genetic aspects of inflammation and immune response in stroke is very dynamic and versatile, and opens numerous options for crosstalk between the basic science and a clinical applications in stroke management and treatment.
